# Supplementary material for: RNA Sequencing (RNA-Seq) Analysis Reveals Liver Lipid Metabolism Divergent Adaptive Response to Low- and High-Salinity Stress in Spotted Scat (Scatophagus argus)
Source: Animals (Basel). 2023 Apr 28;13(9):1503. doi: 10.3390/ani13091503 (PMC10177406; doi:10.3390/ani13091503)
Supplement: Supplementary file 1 [file animals-13-01503-s001.zip › Figure S1 and S2.pdf]

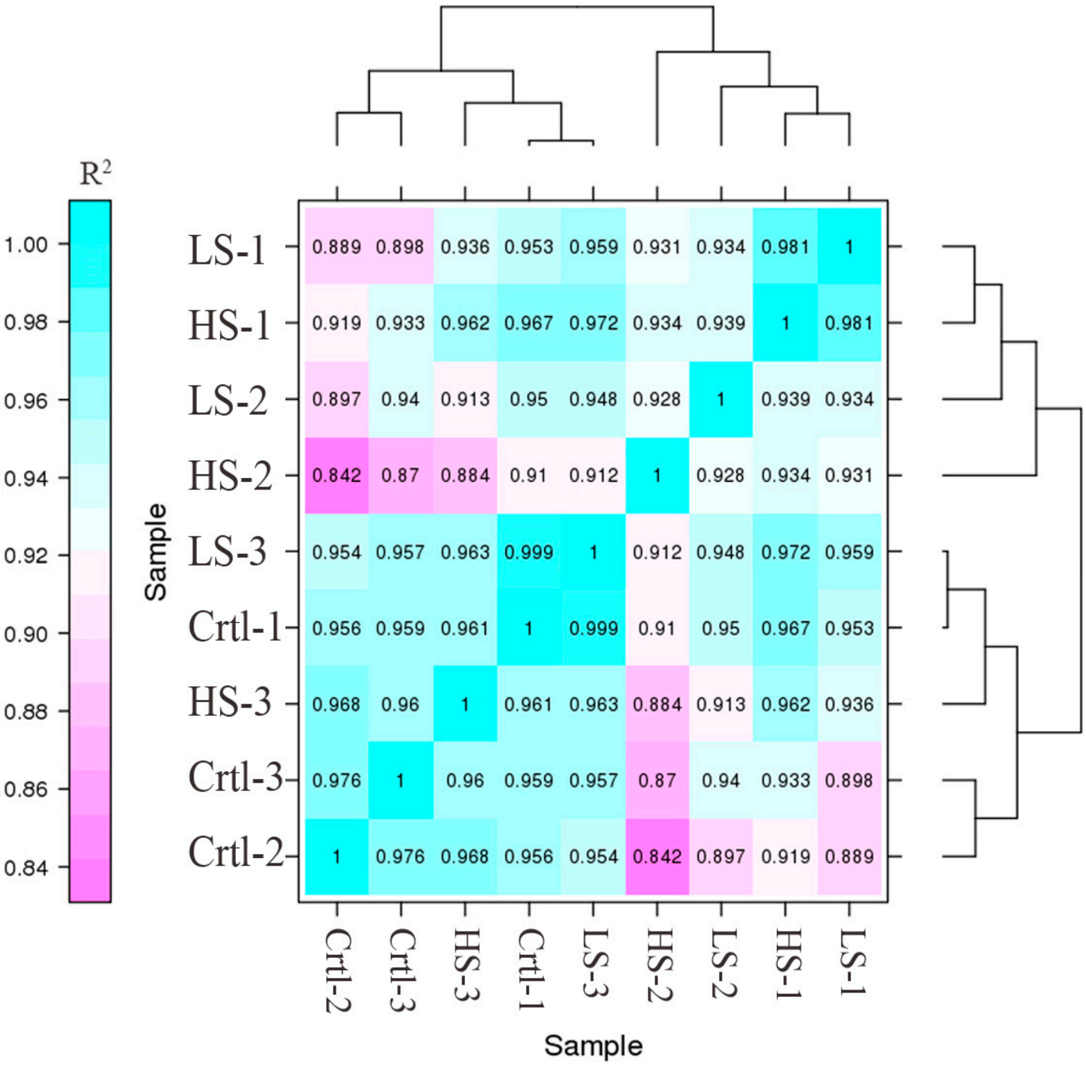

Figure S1. Heat map of pearson correlation coefficients for gene expression levels of liver samples challenge with low- and high in spotted scat. The legend on the left depicts the correlation color scale.

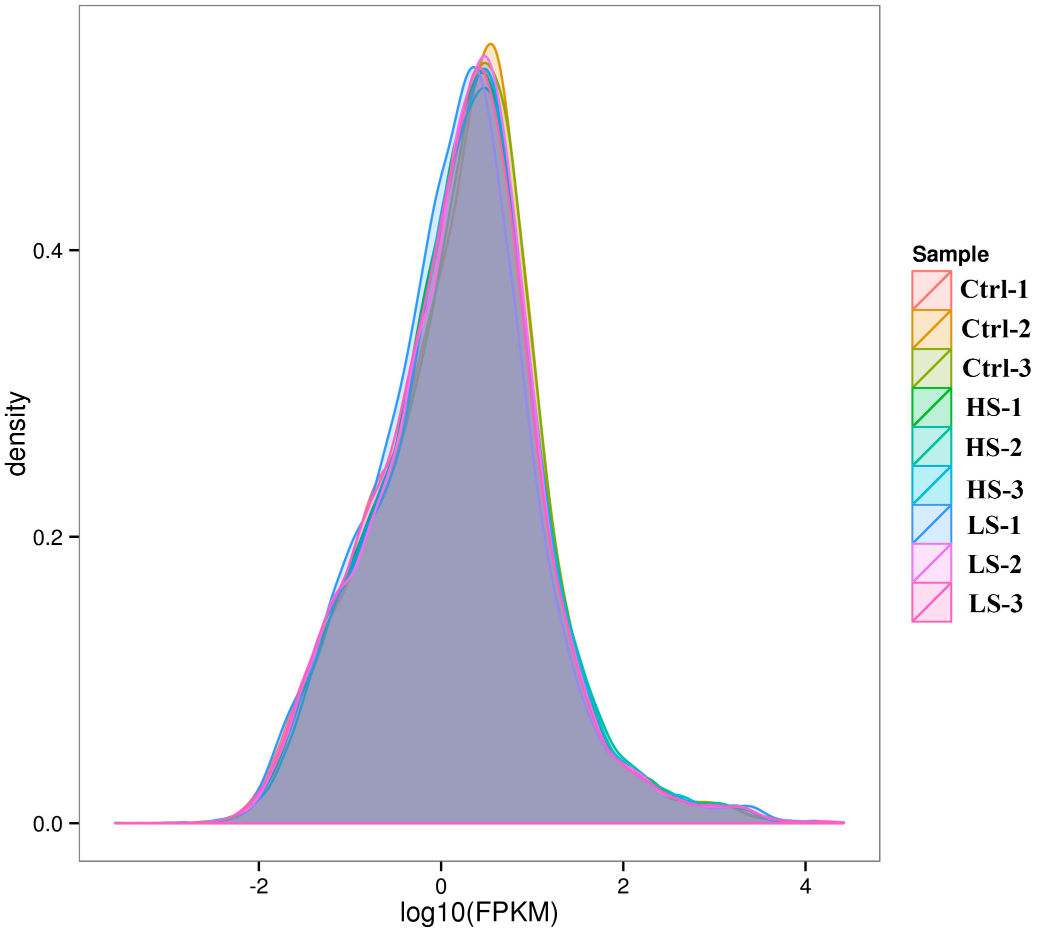

Figure S2. Histogram of FPKM in liver challenge with low- and high salinity in spotted scat.
