# Supplementary material for: RNA Sequencing (RNA-Seq) Analysis Reveals Liver Lipid Metabolism Divergent Adaptive Response to Low- and High-Salinity Stress in Spotted Scat (Scatophagus argus)
Source: Animals (Basel). 2023 Apr 28;13(9):1503. doi: 10.3390/ani13091503 (PMC10177406; doi:10.3390/ani13091503)
Supplement: Supplementary file 1 [file animals-13-01503-s001.zip › Table S1.pdf]

Table S1. The up- and down-regulated KEGG pathways in liver challenge with low- and high salinity in spotted scat

**Up-regulated KEGG**							
**Groups**	**Kegg_pathway**	**ko_id**	**Cluter frequency**	**Genome frequency**	***P*-value**	**Corrected *P*-value [a]**	**-log10 (*P*-value)**
5ppt	Oxidative phosphorylation	ko00190	48/344 13.9534%	141/6606 2.1344%	0.0000	0.0000	7.8327
5ppt	Ribosome	ko03010	87/344 25.2906%	134/6606 2.0284%	0.0000	0.0000	1.6422
5ppt	Proteasome	ko03050	13/344 3.7790%	53/6606 0.8023%	0.0000	0.0003	5.6799
5ppt	Glutathione metabolism	ko00480	11/344 3.1976%	63/6606 0.9536%	0.0003	0.0446	1.5351
5ppt	Metabolism of xenobiotics by cytochrome P450	ko00980	7/344 2.0348%	50/6606 0.7568%	0.0141	1.0000	1.8502
5ppt	Drug metabolism - cytochrome P450	ko00982	7/344 2.0348%	50/6606 0.7568%	0.0141	1.0000	1.8502
5ppt	Cardiac muscle contraction	ko04260	12/344 3.4883%	119/6606 1.8014%	0.0206	1.0000	8.8447
5ppt	Salmonella infection	ko05132	10/344 2.9069%	92/6606 1.3926%	0.0207	1.0000	3.4577
5ppt	Aminoacyl-tRNA biosynthesis	ko00970	6/344 1.7441%	43/6606 0.6509%	0.0228	1.0000	1.6868
5ppt	Protein export	ko03060	4/344 1.1627%	23/6606 0.3481%	0.0292	1.0000	1.6836
35ppt	Fatty acid metabolism	ko01212	17/419 4.0572%	59/6606 0.8931%	0.0000	0.0000	7.1330
35ppt	Proteasome	ko03050	16/419 3.8186%	53/6606 0.8023%	0.0000	0.0000	7.0611
35ppt	Fatty acid biosynthesis	ko00061	9/419 2.1479%	18/6606 0.2724%	0.0000	0.0001	6.3524
35ppt	Peroxisome	ko04146	19/419 4.5346%	97/6606 1.4683%	0.0000	0.0012	5.0914
35ppt	Porphyrin and chlorophyll metabolism	ko00860	10/419 2.3866%	41/6606 0.6206%	0.0002	0.0272	3.7479
35ppt	PPAR signaling pathway	ko03320	15/419 3.5799%	83/6606 1.2564%	0.0002	0.0287	3.7233
35ppt	Glutathione metabolism	ko00480	12/419 2.8639%	63/6606 0.9536%	0.0005	0.0759	3.3013
35ppt	Adipocytokine signaling pathway	ko04920	17/419 4.0572%	110/6606 1.6651%	0.0005	0.0770	3.2953

35ppt	Drug metabolism - cytochrome P450	ko00982	10/419 2.3866%	50/6606 0.7568%	0.0010	0.1489	3.0090
35ppt	Metabolism of xenobiotics by cytochrome P450	ko00980	10/419 2.3866%	50/6606 0.756887677868604%	0.0010	0.1489	3.0090
35ppt	Fatty acid degradation	ko00071	10/419 2.3866%	51/6606 0.7720%	0.0012	0.1749	2.9390
35ppt	Insulin signaling pathway	ko04910	23/419 5.4892%	189/6606 2.8610%	0.0018	0.2778	2.7381
35ppt	Biosynthesis of unsaturated fatty acids	ko01040	7/419 1.6706%	34/6606 0.5146%	0.0047	0.7193	2.3249
35ppt	Ascorbate and aldarate metabolism	ko00053	6/419 1.4319%	29/6606 0.4389%	0.0086	1.0000	2.0678
35ppt	Pyruvate metabolism	ko00620	8/419 1.9093%	47/6606 0.7114%	0.0086	1.0000	2.0633
35ppt	Starch and sucrose metabolism	ko00500	9/419 2.1479%	57/6606 0.8628%	0.0090	1.0000	2.0443
35ppt	Synthesis and degradation of ketone bodies	ko00072	3/419 0.7159%	8/6606 0.1211%	0.0112	1.0000	1.9523
35ppt	Oxidative phosphorylation	ko00190	16/419 3.8186%	141/6606 2.1344%	0.0163	1.0000	1.7866
35ppt	Propanoate metabolism	ko00640	6/419 1.4319%	34/6606 0.5146%	0.0185	1.0000	1.7318
35ppt	Base excision repair	ko03410	6/419 1.4319%	35/6606 0.5298%	0.0212	1.0000	1.6732
35ppt	Amino sugar and nucleotide sugar metabolism	ko00520	9/419 2.1479%	66/6606 0.9990%	0.0225	1.0000	1.6474
35ppt	Valine, leucine and isoleucine degradation	ko00280	8/419 1.9093%	56/6606 0.8477%	0.0238	1.0000	1.6234
35ppt	Galactose metabolism	ko00052	7/419 1.6706%	46/6606 0.6963%	0.0245	1.0000	1.6105
35ppt	Arginine and proline metabolism	ko00330	8/419 1.9093%	58/6606 0.8779%	0.0288	1.0000	1.5408
35ppt	Drug metabolism - other enzymes	ko00983	7/419 1.6706%	49/6606 0.7417%	0.0335	1.0000	1.4753
35ppt	Systemic lupus erythematosus	ko05322	2/419 0.4773%	5/6606 0.0756%	0.0353	1.0000	1.4521
35ppt	Ribosome	ko03010	14/419 3.3412%	134/6606 2.0284%	0.0439	1.0000	1.3571
35ppt	Glycine, serine and threonine metabolism	ko00260	7/419 1.6706%	52/6606 0.7871%	0.0444	1.0000	1.3528
35ppt	Butanoate metabolism	ko00650	4/419	22/6606	0.0470	1.0000	1.3281

			0.9546%	0.3330%			

Down-regulated KEGG

Groups	Kegg_pathway	ko_id	Cluter frequency	Genome frequency	*P*-value	Corrected *P*-value	-log10 (*P*-value)
5ppt	Drug metabolism - other enzymes	ko00983	9/168 5.3571%	49/6606 0.7417%	0.0000	0.0003	5.5115
5ppt	Retinol metabolism	ko00830	9/168 5.3571%	64/6606 0.9688%	0.0000	0.0033	4.5264
5ppt	Ascorbate and aldarate metabolism	ko00053	6/168 3.5714%	29/6606 0.4389%	0.0001	0.0080	4.1409
5ppt	Starch and sucrose metabolism	ko00500	8/168 4.7619%	57/6606 0.8628%	0.0001	0.0094	4.0725
5ppt	Drug metabolism - cytochrome P450	ko00982	7/168 4.1666%	50/6606 0.7568%	0.0002	0.0270	3.6175
5ppt	Porphyrin and chlorophyll metabolism	ko00860	6/168 3.5714%	41/6606 0.6206%	0.0005	0.0595	3.2745
5ppt	Pentose and glucuronate interconversions	ko00040	6/168 3.5714%	43/6606 0.6509%	0.0007	0.0774	3.1605
5ppt	Metabolism of xenobiotics by cytochrome P450	ko00980	6/168 3.5714%	50/6606 0.7568%	0.0016	0.1742	2.8081
5ppt	Steroid hormone biosynthesis	ko00140	6/168 3.5714%	53/6606 0.8023%	0.0021	0.2364	2.6756
5ppt	Steroid biosynthesis	ko00100	4/168 2.3809%	24/6606 0.3633%	0.0029	0.3227	2.5405
5ppt	Glycerophospholipid metabolism	ko00564	9/168 5.3571%	119/6606 1.8013%	0.0032	0.3549	2.4991
5ppt	Glycerolipid metabolism	ko00561	7/168 4.16666%	81/6606 1.2261%	0.0043	0.4868	2.3619
5ppt	Pantothenate and CoA biosynthesis	ko00770	3/168 1.7857%	15/6606 0.2270%	0.0059	0.6573	2.2314
5ppt	Phosphatidylinositol signaling system	ko04070	8/168 4.7619%	133/6606 2.0133%	0.0197	1	1.7053
5ppt	Spliceosome	ko03040	8/168 4.7619%	142/6606 2.1495%	0.0279	1	1.5544
5ppt	Lysine degradation	ko00310	5/168 2.9761%	72/6606 1.0899%	0.0357	1	1.4469
5ppt	Ribosome biogenesis in eukaryotes	ko03008	5/168 2.9761%	77/6606 1.1656%	0.0457	1	1.3403
35ppt	Ribosome biogenesis in eukaryotes	ko03008	30/460 6.5217%	77/6606 1.16560%	0.0000	0.0000	12.8297

35ppt	Protein processing in endoplasmic reticulum	ko04141	54/460 11.7391%	204/6606 3.0881%	0.0000	0.0000	11.8665
35ppt	N-Glycan biosynthesis	ko00510	22/460 4.7826%	68/6606 1.0293%	0.0000	0.0000	9.3747
35ppt	Lysine degradation	ko00310	16/460 3.4782%	72/6606 1.0899%	0.0000	0.0035	4.5952
35ppt	RNA transport	ko03013	23/460 5%	160/6606 2.4220%	0.0006	0.0882	3.1881
35ppt	Protein export	ko03060	7/460 1.5217%	23/6606 0.3481%	0.0007	0.0948	3.1567
35ppt	ECM-receptor interaction	ko04512	14/460 3.04347%	107/6606 1.6197%	0.0159	1	1.7992
35ppt	Starch and sucrose metabolism	ko00500	9/460 1.9565%	57/6606 0.8628%	0.0160	1	1.7954
35ppt	Amino sugar and nucleotide sugar metabolism	ko00520	9/460 1.9565%	66/6606 0.9990%	0.0381	1	1.4195
35ppt	RNA degradation	ko03018	11/460 2.3913%	89/6606 1.3472%	0.0439	1	1.3580

a Corrected *P*-value: *P* value adjusted by Benjamini and Hochberg procedure.

Enrichment DEGs potentially associated with lipid metabolism in the liver challenge with different salinity in spotted scat.

**Gene ID**	**Gene name**	**Log$_2$ FoldChange**	***P*-value**	**Gene function**
**LS vs. Ctrl**				
EVM0023995	*plpp1*, ppap2a; phospholipid phosphatase 1	-0.494279179	0.021340592	Glycerolipid metabolism (ko00561);; Glycerophospholipid metabolism (ko00564);; Ether lipid metabolism (ko00565);; Sphingolipid metabolism (ko00600)
EVM0011966	phosphatidylinositol 4-kinase alpha-like	-0.497348647	0.008457581	Inositol phosphate metabolism (ko00562);; Phosphatidylinositol signaling system (ko04070)
EVM0000521	*cept1*; choline/ethanolamine phosphotransferase 1	-0.53137678	0.01666086	Glycerophospholipid metabolism (ko00564);; Ether lipid metabolism (ko00565)
EVM0020842	*pi4kb*; phosphatidylinositol 4-kinase beta	-0.565603672	0.004689426	Inositol phosphate metabolism (ko00562);; Phosphatidylinositol signaling system (ko04070)
EVM0010040	*dhcr7*;	-0.507776896	0.02000388	Steroid biosynthesis (ko00100)

	7-dehydrocholesterol reductase			
EVM0023989	non-specific lipid-transfer protein-like	0.326696072	0.049738268	Primary bile acid biosynthesis (ko00120);; PPAR signaling pathway (ko03320);; Peroxisome (ko04146)
EVM0018902	*dgki*; diacylglycerol kinase iota	-0.632031625	0.008944992	Glycerolipid metabolism (ko00561);; Glycerophospholipid metabolism (ko00564);; Phosphatidylinositol signaling system (ko04070)
EVM0009855	type II inositol 3,4-bisphosphate 4-phosphatase-like	-0.571422947	0.008086221	Inositol phosphate metabolism (ko00562);; Phosphatidylinositol signaling system (ko04070)
EVM0011059	*ebp*; emopamil binding protein (sterol isomerase)	0.491714624	0.041713644	Steroid biosynthesis (ko00100)
EVM0006384	*sqle*; squalene epoxidase	-0.622426552	0.007394244	Steroid biosynthesis (ko00100)
EVM0015017	carnitine O-palmitoyltransferase 1, liver isoform-like	-0.703384516	0.001864054	Fatty acid degradation (ko00071);; Fatty acid metabolism (ko01212);; PPAR signaling pathway (ko03320);; Adipocytokine signaling pathway (ko04920)
EVM0010163	*dgat1*; diacylglycerol O-acyltransferase 1	-0.493431557	0.020757088	Glycerolipid metabolism (ko00561);; Retinol metabolism (ko00830)
EVM0021472	*dgkh*; diacylglycerol kinase eta	-0.416051075	0.049751166	Glycerolipid metabolism (ko00561);; Glycerophospholipid metabolism (ko00564);; Phosphatidylinositol signaling system (ko04070)
EVM0022818	*ptdss2*; phosphatidylserine synthase 2	-0.379058107	0.03491172	Glycerophospholipid metabolism (ko00564)
EVM0020789	choline-phosphate cytidylyltransferase B-like	0.523057443	0.002042466	Glycerophospholipid metabolism (ko00564)
EVM0010560	choline O-acetyltransferase-like	-0.699397739	0.003854584	Glycerophospholipid metabolism (ko00564)
EVM0015933	1-phosphatidylinositol 4,5-bisphosphate phosphodiesterase gamma-1-like	-0.349994567	0.024186643	Inositol phosphate metabolism (ko00562);; ErbB signaling pathway (ko04012);; Calcium signaling pathway (ko04020);; Phosphatidylinositol signaling system (ko04070);; VEGF signaling pathway (ko04370);; AGE-RAGE signaling pathway in diabetic complications (ko04933)

EVM0012014	*pla2g4a*, phospholipase A2-like	-0.802009596	0.000668229	Glycerophospholipid metabolism (ko00564);; Ether lipid metabolism (ko00565);; Arachidonic acid metabolism (ko00590);; Linoleic acid metabolism (ko00591);; alpha-Linolenic acid metabolism (ko00592);; Vascular smooth muscle contraction (ko04270)
EVM0001595	*pde2a*; phosphodiesterase 2A	-0.452091855	0.008012239	Purine metabolism (ko00230)
EVM0006432	diacylglycerol kinase delta-like	-0.440725073	0.044151136	Glycerolipid metabolism (ko00561);; Glycerophospholipid metabolism (ko00564);; Phosphatidylinositol signaling system (ko04070)
EVM0001802	3-keto-steroid reductase-like	0.463262617	0.040933246	Steroid biosynthesis (ko00100);; Steroid hormone biosynthesis (ko00140)
EVM0016821	lipid phosphate phosphohydrolase 1-like	0.464536819	0.029006871	Glycerolipid metabolism (ko00561);; Glycerophospholipid metabolism (ko00564);; Ether lipid metabolism (ko00565);; Sphingolipid metabolism (ko00600)
EVM0013275	*fat-5*; delta-9-desaturase 2	0.508207303	0.028889235	Biosynthesis of unsaturated fatty acids (ko01040);; Fatty acid metabolism (ko01212);; PPAR signaling pathway (ko03320)
EVM0016720	inpp4b; inositol polyphosphate-4-phosphatase type II B	0.51437671	0.027416164	Inositol phosphate metabolism (ko00562);; Phosphatidylinositol signaling system (ko04070)
EVM0000633	*scd1*, delta-9-desaturase 1	0.558057248	0.006520205	Biosynthesis of unsaturated fatty acids (ko01040);; Fatty acid metabolism (ko01212);; PPAR signaling pathway (ko03320)
EVM0023840	*pmm2*; phosphomannomutase 2	0.609101233	0.001365412	Fructose and mannose metabolism (ko00051);; Amino sugar and nucleotide sugar metabolism (ko00520)
EVM0021910	acyl-CoA-binding protein-like	0.550350103	0.01202275	PPAR signaling pathway (ko03320)
EVM0017700	*fabp6*; fatty acid binding protein 6	0.600901202	0.010799413	PPAR signaling pathway (ko03320)
EVM0004065	*fabp3*; fatty acid binding protein 3	0.468866691	0.010276546	PPAR signaling pathway (ko03320)
EVM0009831	*pck1*; phosphoenolpyruvate carboxykinase 1	-0.466918319	0.03953855	Glycolysis / Gluconeogenesis (ko00010);; Citrate cycle (TCA cycle) (ko00020);; Pyruvate metabolism (ko00620);; PPAR signaling pathway (ko03320);; FoxO signaling pathway (ko04068);; Insulin signaling

				pathway (ko04910);; Adipocytokine signaling pathway (ko04920)
EVM0003985	*slc27a4*; solute carrier family 27 member 4	-0.522079923	0.016433613	PPAR signaling pathway (ko03320)
EVM0006371	fatty acid-binding protein, brain-like	0.408421355	0.019377584	PPAR signaling pathway (ko03320)
EVM0009263	*lpl*,lipoprotein lipase-like	-0.420533992	0.024328376	Glycerolipid metabolism (ko00561);; PPAR signaling pathway (ko03320)
EVM0003401	*ugt2a1*, UDP-glucuronosyltransferase 2A1	-0.552813189	0.004898142	Steroid hormone biosynthesis
EVM0003565	*ugt2a2*, UDP-glucuronosyltransferase 2A2-like	-0.466258335	0.032223589	Steroid hormone biosynthesis
EVM0005100	*ugt2b31*, UDP-glucuronosyltransferase 2B31-like	-0.871147633	9.77E-05	Steroid hormone biosynthesis
EVM0019647	*ugt2b20*, UDP-glucuronosyltransferase 2B20-like	-0.457260075	0.020732666	Steroid hormone biosynthesis
EVM0023884	cyp51a1, lanosterol 14-alpha demethylase	-0.625469837	0.009212617	Steroid biosynthesis
**HS vs. Ctrl**				
EVM0005987	long-chain-fatty-acid--CoA ligase ACSBG2-like	2.128430956	5.74E-22	Fatty acid biosynthesis (ko00061);; Fatty acid degradation (ko00071);; Fatty acid metabolism (ko01212);; PPAR signaling pathway (ko03320);; Adipocytokine signaling pathway (ko04920)
EVM0017477	long-chain-fatty-acid--CoA ligase ACSBG2-like	2.003194766	1.45E-18	Fatty acid biosynthesis (ko00061);; Fatty acid degradation (ko00071);; Fatty acid metabolism (ko01212);; PPAR signaling pathway (ko03320);; Adipocytokine signaling pathway (ko04920)
EVM0000633	*scd1*, delta-9-desaturase 1	1.468072624	9.61E-08	Biosynthesis of unsaturated fatty acids (ko01040);; Fatty acid metabolism (ko01212);; PPAR signaling pathway (ko03320)
EVM0019818	long-chain-fatty-acid--CoA ligase 3	1.355983665	4.23E-10	Fatty acid biosynthesis (ko00061);; Fatty acid degradation (ko00071);; Fatty acid metabolism

				(ko01212);; PPAR signaling pathway (ko03320);; Peroxisome (ko04146);; Adipocytokine signaling pathway (ko04920)
EVM0009139	*fas*, fatty acid synthase	1.243001461	4.06E-09	Fatty acid biosynthesis (ko00061);; Fatty acid metabolism (ko01212);; Insulin signaling pathway (ko04910)
EVM0023488	*acaca*; acetyl-CoA carboxylase alpha	1.185429522	2.97E-11	Fatty acid biosynthesis (ko00061);; Pyruvate metabolism (ko00620);; Propanoate metabolism (ko00640);; Fatty acid metabolism (ko01212);; Insulin signaling pathway (ko04910)
EVM0019959	*acsl4*; acyl-CoA synthetase long-chain family member 4	1.070217899	1.18E-05	Fatty acid biosynthesis (ko00061);; Fatty acid degradation (ko00071);; Fatty acid metabolism (ko01212);; PPAR signaling pathway (ko03320);; Peroxisome (ko04146);; Adipocytokine signaling pathway (ko04920)
EVM0020874	*fads2*, fatty acid desaturase 2-like	0.938107743	0.000187633	alpha-Linolenic acid metabolism (ko00592);; Biosynthesis of unsaturated fatty acids (ko01040);; Fatty acid metabolism (ko01212);; PPAR signaling pathway (ko03320)
EVM0017784	*acsbg2*; acyl-CoA synthetase bubblegum family member 2	0.894931383	0.000445813	Fatty acid biosynthesis (ko00061);; Fatty acid degradation (ko00071);; Fatty acid metabolism (ko01212);; PPAR signaling pathway (ko03320);; Adipocytokine signaling pathway (ko04920)
EVM0013275	*fat-5*; delta-9-desaturase 2	0.861686751	0.009356233	Biosynthesis of unsaturated fatty acids (ko01040);; Fatty acid metabolism (ko01212);; PPAR signaling pathway (ko03320)
EVM0011010	*agpat2*; 1-acyl-sn-glycerol-3-phosphate acyltransferase alpha	0.849321916	8.34E-05	Glycerolipid metabolism (ko00561);; Glycerophospholipid metabolism (ko00564)
EVM0006371	fatty acid-binding protein, brain-like	0.834042122	4.20E-07	PPAR signaling pathway (ko03320)
EVM0019411	*acsl3*; acyl-CoA synthetase long-chain family member 3	0.816404445	0.005575647	Fatty acid biosynthesis (ko00061);; Fatty acid degradation (ko00071);; Fatty acid metabolism (ko01212);; PPAR signaling pathway (ko03320);; Peroxisome (ko04146);; Adipocytokine signaling pathway (ko04920)
EVM0006208	*pcyt1a*; phosphate cytidylyltransferase 1, choline, alpha	0.759544129	4.26E-06	Glycerophospholipid metabolism (ko00564)
EVM0021376	*gpam*;	0.717124628	0.002697694	Glycerolipid metabolism (ko00561);;

	glycerol-3-phosphate acyltransferase, mitochondrial			Glycerophospholipid metabolism (ko00564)
EVM0003100	*ptdss1*; phosphatidylserine synthase 1	0.665992365	0.013819658	Glycerophospholipid metabolism (ko00564)
EVM0023042	*acsl6*; long-chain-fatty-acid--CoA ligase 6	0.662029952	0.001552236	Fatty acid biosynthesis (ko00061);; Fatty acid degradation (ko00071);; Fatty acid metabolism (ko01212);; PPAR signaling pathway (ko03320);; Peroxisome (ko04146);; Adipocytokine signaling pathway (ko04920)
EVM0023989	non-specific lipid-transfer protein-like	0.569565768	0.000391749	Primary bile acid biosynthesis (ko00120);; PPAR signaling pathway (ko03320);; Peroxisome (ko04146)
EVM0023716	*elovl6*; ELOVL fatty acid elongase 6	0.553695318	0.009147	Fatty acid elongation (ko00062);; Biosynthesis of unsaturated fatty acids (ko01040);; Fatty acid metabolism (ko01212)
EVM0007369	*acat2*; acetyl-CoA acetyltransferase 2	0.544407427	0.022523644	Fatty acid degradation (ko00071);; Synthesis and degradation of ketone bodies (ko00072);; Valine, leucine and isoleucine degradation (ko00280);; Lysine degradation (ko00310);; Tryptophan metabolism (ko00380);; Pyruvate metabolism (ko00620);; Glyoxylate and dicarboxylate metabolism (ko00630);; Propanoate metabolism (ko00640);; Butanoate metabolism (ko00650);; Terpenoid backbone biosynthesis (ko00900);; Carbon metabolism (ko01200);; Fatty acid metabolism (ko01212)
EVM0002066	elongation of very long chain fatty acids protein 4-like	0.520315327	0.024456114	Fatty acid elongation (ko00062)
EVM0023840	*pmm2*; phosphomannomutase 2	0.515123346	0.003679859	Fructose and mannose metabolism (ko00051);; Amino sugar and nucleotide sugar metabolism (ko00520)
EVM0000962	*alg10*; ALG10, alpha-1,2-glucosyltransferase	0.489851737	0.036929631	N-Glycan biosynthesis (ko00510)
EVM0002080	*elovl1*; ELOVL fatty acid elongase 1	0.451575173	0.006644309	Fatty acid elongation (ko00062);; Biosynthesis of unsaturated fatty acids (ko01040);; Fatty acid metabolism (ko01212)
EVM0023922	*soat2*; sterol O-acyltransferase 1	0.442393311	0.02647554	Steroid biosynthesis (ko00100)
EVM0020789	choline-phosphate cytidylyltransferase	0.385388294	0.018090583	Glycerophospholipid metabolism (ko00564)

	B-like			
EVM0012062	very long-chain acyl-CoA synthetase-like	0.360053852	0.040075358	PPAR signaling pathway (ko03320);; Peroxisome (ko04146)
EVM0006069	*pecr*; peroxisomal trans-2-enoyl-CoA reductase	0.381801622	0.04125754	Biosynthesis of unsaturated fatty acids (ko01040);; Fatty acid metabolism (ko01212);; Peroxisome (ko04146)
EVM0004548	diacylglycerol O-acyltransferase 2-like	0.353650797	0.032354179	Glycerolipid metabolism (ko00561)
EVM0016407	*hadha*; hydroxyacyl-CoA dehydrogenase/3-ketoacyl-CoA thiolase/enoyl-CoA hydratase (trifunctional protein), alpha subunit	-0.3019208	0.043027279	Fatty acid elongation (ko00062);; Fatty acid degradation (ko00071);; Valine, leucine and isoleucine degradation (ko00280);; Lysine degradation (ko00310);; Tryptophan metabolism (ko00380);; beta-Alanine metabolism (ko00410);; Propanoate metabolism (ko00640);; Butanoate metabolism (ko00650);; Biosynthesis of unsaturated fatty acids (ko01040);; Carbon metabolism (ko01200);; Fatty acid metabolism (ko01212)
EVM0009357	*acadl*; acyl-CoA dehydrogenase, long chain	-0.336969516	0.048431814	Fatty acid degradation (ko00071);; Fatty acid metabolism (ko01212);; PPAR signaling pathway (ko03320)
EVM0016188	*acads*; acyl-CoA dehydrogenase, C-2 to C-3 short chain	-0.405413102	0.030954522	Fatty acid degradation (ko00071);; Valine, leucine and isoleucine degradation (ko00280);; Butanoate metabolism (ko00650);; Carbon metabolism (ko01200);; Fatty acid metabolism (ko01212)
EVM0022355	*hadh*; hydroxyacyl-CoA dehydrogenase	-0.453193053	0.002455633	Fatty acid elongation (ko00062);; Fatty acid degradation (ko00071);; Valine, leucine and isoleucine degradation (ko00280);; Lysine degradation (ko00310);; Tryptophan metabolism (ko00380);; Butanoate metabolism (ko00650);; Fatty acid metabolism (ko01212)
EVM0001050	ethanolamine-phosphate cytidylyltransferase-like	-0.469341032	0.040281287	Glycerophospholipid metabolism (ko00564)
EVM0002240	*dgkd*; diacylglycerol kinase delta	-0.47997202	0.00919679	Glycerolipid metabolism (ko00561);; Glycerophospholipid metabolism (ko00564);; Phosphatidylinositol signaling system (ko04070)
EVM0008093	glycerol-3-phosphate acyltransferase	-0.481791319	0.003975881	Glycerolipid metabolism (ko00561);; Glycerophospholipid metabolism (ko00564)

	3-like			
EVM0010884	phosphoethanolamine N-methyltransferase 3-like	-0.495882452	0.002226424	Glycerophospholipid metabolism (ko00564)
EVM0012014	*pla2g4a*; phospholipase A2-like	-0.548207575	0.047685744	Glycerophospholipid metabolism (ko00564);; Ether lipid metabolism (ko00565);; Arachidonic acid metabolism (ko00590);; Linoleic acid metabolism (ko00591);; alpha-Linolenic acid metabolism (ko00592);; Vascular smooth muscle contraction (ko04270)
EVM0018220	*cds1*; phosphatidate cytidylyltransferase 2	-0.557450912	0.009661068	Glycerophospholipid metabolism (ko00564);; Phosphatidylinositol signaling system (ko04070)
EVM0010163	*dgat1*; diacylglycerol O-acyltransferase 1	-0.691099148	0.002635581	Glycerolipid metabolism (ko00561);; Retinol metabolism (ko00830)
EVM0000521	*cept1*; choline/ethanolamine phosphotransferase 1	-0.709767922	0.006670652	Glycerophospholipid metabolism (ko00564);; Ether lipid metabolism (ko00565)
EVM0022170	*msmo1*; methylsterol monooxygenase 1	-0.792119843	0.000251552	Steroid biosynthesis (ko00100)
EVM0006432	diacylglycerol kinase delta-like	-0.990811261	0.000274616	Glycerolipid metabolism (ko00561);; Glycerophospholipid metabolism (ko00564);; Phosphatidylinositol signaling system (ko04070)
EVM0002904	angiopoietin-related protein 4-like	-0.727623207	0.003544393	PPAR signaling pathway (ko03320)
EVM0006506	*angptl4*; angiopoietin like 4	-0.766395737	0.006458709	PPAR signaling pathway (ko03320)
EVM0023042	*acsl6*; long-chain-fatty-acid--CoA ligase 6	0.662029952	0.001552236	Fatty acid biosynthesis (ko00061);; Fatty acid degradation (ko00071);; Fatty acid metabolism (ko01212);; PPAR signaling pathway (ko03320);; Peroxisome (ko04146);; Adipocytokine signaling pathway (ko04920)
EVM0009831	*pck1*; phosphoenolpyruvate carboxykinase 1	0.518387425	0.047957258	Glycolysis / Gluconeogenesis (ko00010);; Citrate cycle (TCA cycle) (ko00020);; Pyruvate metabolism (ko00620);; PPAR signaling pathway (ko03320);; FoxO signaling pathway (ko04068);; Insulin signaling

				pathway (ko04910);; Adipocytokine signaling pathway (ko04920)
EVM0021145	*ppara*; peroxisome proliferator activated receptor alpha	0.481586273	0.048252519	PPAR signaling pathway (ko03320);; Adipocytokine signaling pathway (ko04920)
EVM0022179	sorbin and SH3 domain-containing protein 1-like	-0.501791766	0.003442684	PPAR signaling pathway (ko03320);; Adherens junction (ko04520);; Insulin signaling pathway (ko04910)
EVM0001796	*g6pc1*, glucose-6-phosphatase	1.173745732	0.000481306	Adipocytokine signaling pathway (ko04920)
EVM0001859	*socs1*, suppressor of cytokine signaling	1.179125942	0.000450826	Adipocytokine signaling pathway (ko04920)
EVM0003316	*socs3*, suppressor of cytokine signaling 3	1.313341782	1.90E-07	Adipocytokine signaling pathway (ko04920)
EVM0006476	Adipor2, adiponectin receptor protein 2	0.430474726	0.008397291	Adipocytokine signaling pathway (ko04920)
